# Oral administration of withaferin A inhibits carcinogenesis of prostate in TRAMP model

**DOI:** 10.18632/oncotarget.10733

**Published:** 2016-07-20

**Authors:** Suman Suman, Trinath P. Das, Jim Moselhy, Deeksha Pal, Venkatesh Kolluru, Houda Alatassi, Murali K. Ankem, Chendil Damodaran

**Affiliations:** ^1^ Department of Urology, University of Louisville, KY, USA; ^2^ Department of Pathology, University of Louisville, KY, USA

**Keywords:** dietary agents, chemoprevention, prostate cancer

## Abstract

We previously reported that withaferin A (WA), a natural compound, deters prostate cancer by inhibiting AKT while inducing apoptosis. In the current study, we examined its chemopreventive efficacy against carcinogenesis in the prostate using the transgenic adenocarcinoma of mouse prostate (TRAMP) model. Two distinct sets of experiments were conducted. To determine whether WA delays tumor progression, it was given before cancer onset, at week 6, and until week 44. To determine its effect after the onset of prostate cancer, it was given from weeks 12 to 35. In both strategies, oral administration of WA effectively suppressed tumor burden when compared to vehicle-treated animals. No toxicity was seen in treated animals at gross pathological examination. Western blot analysis and immunohistochemistry of tumor sections revealed that in TRAMP controls, AKT and pAKT were highly expressed while nuclear FOXO3a and Par-4 were downregulated. On the contrary, treated mice showed inhibition of AKT signaling and activation of FOX03a-Par-4-induced cell death. They also displayed inhibition of mesenchymal markers such as β-catenin, vimentin, and snail as well as upregulation of E-cadherin. Because expressions of the angiogenic markers factor VIII and retic were downregulated, an anti-angiogenic role of WA is suggested. Overall, our results suggest that WA could be a promising anti-cancer agent that effectively inhibits carcinogenesis of the prostate.

## INTRODUCTION

Finding a treatment option for advanced prostate cancer is challenging, and at this stage, chemotherapy and androgen ablation therapy provide limited success. In general, most prostate cancer patients respond to most of the current treatment options; however, once the disease progresses to castration-resistant prostate cancer (CRPC), limited options are available [1–3]. Novel therapeutic targets are being identified and potent molecules are being developed to either prevent disease progression or treat it after onset. Aberrant activation of the androgen receptor (AR) is a major driving force for carcinogenesis in the prostate; 5- alpha-reductase inhibitors such as distasteride and finasteride disrupt the conversion of testosterone to dihydrotestosterone, there by inhibiting AR and preventing disease progression [4, 5]. Alternatively, diet-based agents such as lycopene and isoflavone have shown promising results in clinical trials due in part to lowering of testosterone levels and a more favorable estrogen/testosterone balance in prostate cancer patients [6–8]. Thus, establishing the efficacy and tolerance of dietary agents in preclinical models of the disease is important for the development of alternative prostate cancer treatment strategies.

A number of molecular targets have been identified in CRPC, one of which is AKT [9]. We [10] and others have reported a gradual increase in nuclear pAKT expression in various stages of prostate cancer (Gleason scores between 6 and 10), resulting in chemoresistance and a possible reason for CRPC [11–13]. The tumor suppressor PTEN negatively regulates AKT function; however, mutation in (or deletion of) PTEN either in primary (30%) or metastatic (63%) prostate tumors is often responsible for AKT-mediated survival signaling [14], resulting in downregulation of pro-apoptotic signaling governed by FOXO3a and Par-4 [15–17]. Hence, inhibiting AKT has been considered an attractive objective, and recently, AKT inhibitors have been tested in clinical trials.

In recent years, both scientists and patients have had great interests in using dietary agents in preventive strategies against prostate cancer. Randomized trials designed to determine the protective effect of vitamin E showed that it is promising against prostate cancer [18–20]. Similarly, studies of pomegranate extract further encouraged prostate cancer patients to adapt to complementary and alternative strategies for preventing the disease progression. Ample epidemiological studies clearly demonstrate the beneficial effects of dietary agents on prostate cancer. Withaferin A (WA) is one such dietary agent. Present in *Withania sominefera*, it has been extensively used in Asian and African traditional medicine for treating various types of cancers, inflammation, and neurological disorders [21, 22]. It has been shown to cause cytotoxicity in various cancer types [23, 24] including prostate cancer. We have previously demonstrated that the pro-apoptotic protein, Par-4, is targeted by WA, and this is how it executes its anticancer effect against CRPC [25]. Very recently, we have shown that AKT negatively regulates Par-4 function by phosphorylating FOXO3a at ser253. Inhibition of AKT by WA reverts the FOXO3a/Par-4-mediated pro-apoptotic function in CRPC, and this was further confirmed through xenograft studies [10]. However, the chronic toxicity of WA and its preventive effect on the primary carcinogenesis of prostate cancer have yet to be established. Therefore, in this study, we evaluated the chemopreventive effects of WA on transgenic adenocarcinoma of mouse prostate (TRAMP) mice, the ideal model for prostate cancer chemoprevention [26–28]. Because there are many similarities between human and TRAMP prostate cancers, this model is considered an appropriate model for evaluating chemopreventive and/or therapeutic strategies against prostate cancer [29–34].

In this study, we showed that WA significantly inhibited prostate adenocarcinoma in TRAMP transgenic mice. It's *in vivo* mechanisms of action confirmed inhibition of pAKT expression, facilitating FOXO3a/Par-4-mediated tumor inhibition in TRAMP mice.

## RESULTS

### Oral administration of WA inhibits prostate tumorigenesis and metastasis in TRAMP mice

Age-dependent prostate growth is defined in the TRAMP model: high-grade PIN occurs at 6 to 8 weeks, well differentiated carcinoma at 8 to 14 weeks, poorly differentiated adenocarcinoma at 16 to 20 weeks, and metastasis at 20 weeks and beyond. Taking advantage of this, two distinct sets of experiments were conducted, one to determine whether dietary WA delays tumor progression when given from weeks 6 to 44, and the other to examine the effect of WA after prostate cancer onset. The WA was given by oral gavage, and control mice received sesame oil throughout the experimental period.

**Figure 1:**
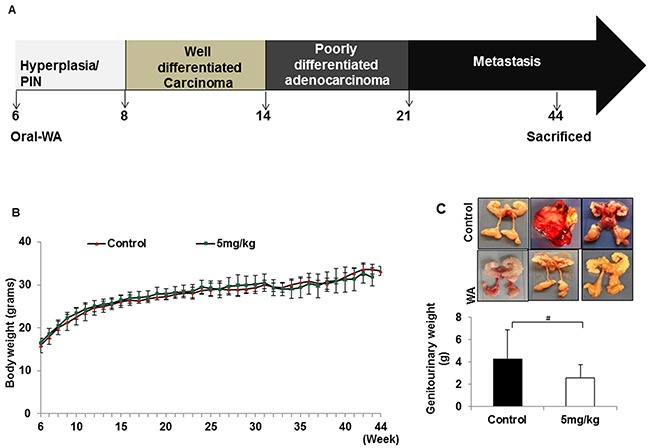
**A.** Diagrammatic representation of TRAMP mouse prostate carcinogenesis and course of study. **B.** All mice were weighed weekly, and the line graph represents average body weight. **C.** Mice treated with WA (5mg/kg) or vehicle was sacrificed after 44 weeks and the genitourinary systems were extracted and weighed.

In the first set of experiments, one of two concentrations of WA (3 or 5 mg/kg body weight) was given to the treatment group starting at week 6, and animals were examined for prostate tumor growth at 10, 20, 30, 40, and 45 weeks of age (Figure 1A). In both cases, no adverse toxic effects were found in any organs over the 38-week period, similar to that seen in control mice (Figure 1B). However, the genitourinary organs weighed 4.23 g in control and 3.15 g in treated mice, suggesting inhibition of tumor burden by WA-treatment (Figure 1C).

**Figure 2:**
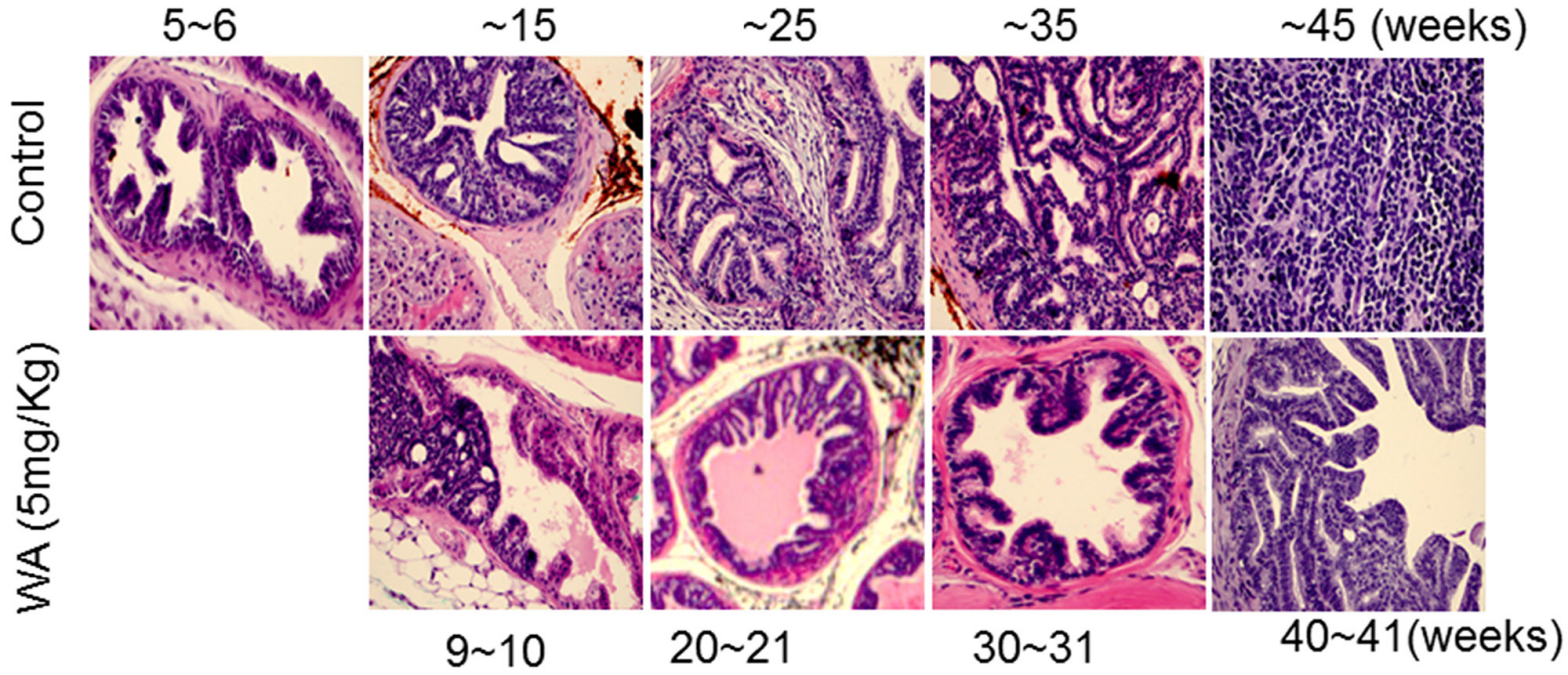
Control and WA-treated mice were periodically sacrificed, and prostate tumor tissues were subjected to H&E analysis

Pathological studies in control mice suggested high-grade PIN at week 6 and also at week 11-12, but carcinoma at week 20. The carcinoma became more aggressive and less differentiated beginning of week 29 and neuroendocrine/aggressive carcinoma noted later (Figure 2). At the higher WA dose (5mg/kg), 9 to 10 weeks of treatment prevented the development of adenocarcinoma, and all mice showed high-grade PIN, which persisted until week 29, (i.e) after 17 weeks of treatment. At termination (week 34), no high grade carcinoma or aggressive neuroendocrine tumor was noted in WA-treated mice (Figure 2).

**Figure 3:**
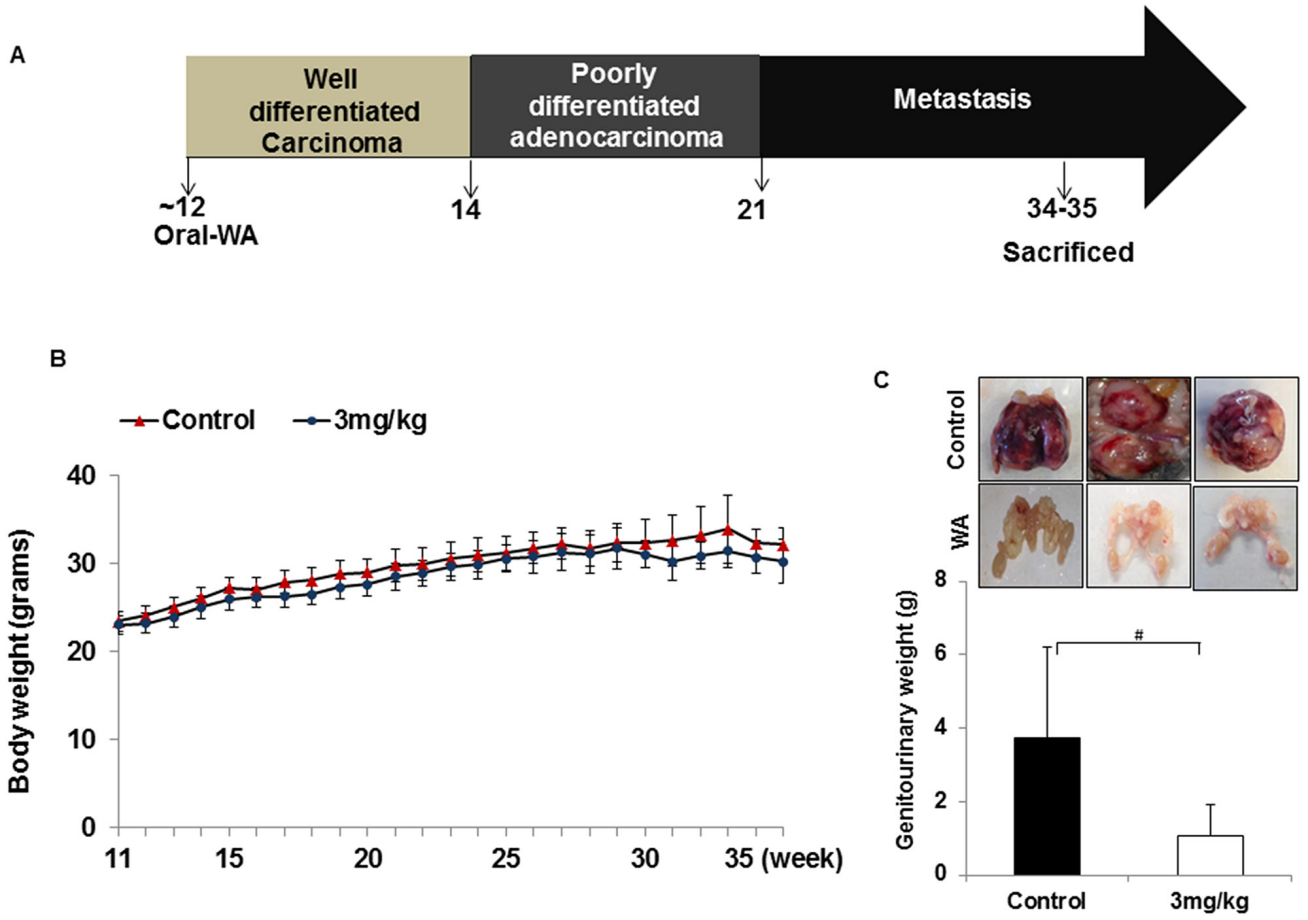
**A.** Diagrammatic representation of TRAMP mouse prostate carcinogenesis and course of study. **B.** All mice were weighed weekly, and the line graph represents average body weight. **C.** Mice treated with WA (3mg/kg) or vehicle were sacrificed after 34 weeks and the genitourinary systems were extracted and weighed.

**Figure 4:**
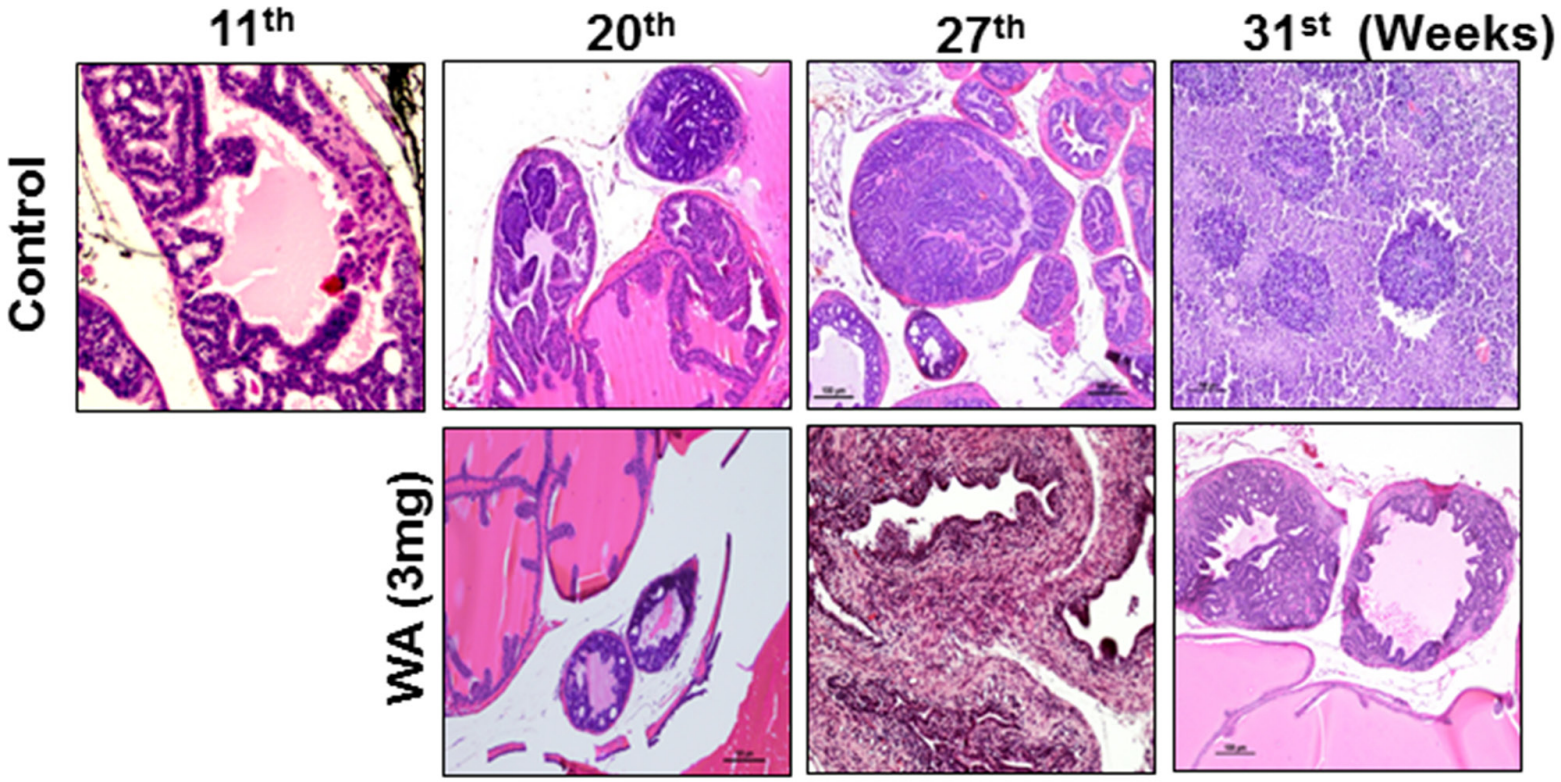
Images for mouse prostate tissues after H&E staining Control and WA-treated mice were periodically sacrificed, and prostate tumor tissues were subjected to H&E analysis.

In the second set of experiments, WA was given at 3 mg/kg beginning at the age of 11 to 12 weeks, after the onset of high-grade PIN, and until the age of 34 weeks (23-24 weeks of treatment). No significant differences in body weight was found between controls and treatment groups (Figure 3B). Genitourinary organs weighed 3.75 g in controls and 1.05 g in treated mice (Figure 3C). Pathological studies in control mice suggested high-grade PIN at weeks 11-12, carcinoma at week 20, and neuroendocrine/aggressive carcinoma beginning at week 29. Only one of 12 controls did not develop a tumor, while all others had either invasive carcinoma or neuroendocrine tumors (Figure 4). Oral administration of WA for 8 to 9 weeks prevented the development of adenocarcinoma, and all mice showed benign changes until week 29 (17 weeks of treatment). At termination (week 34), one mouse showed normal pathology, 2 had high-grade PIN, 1 had possible endocrine tumor morphology, and the remaining three had pathologies similar to high grade PIN. Interestingly, WA significantly inhibited the bulk of the tumor burden until termination (Figure 4). Up to week 29, H&E staining indicated no metastasis in WA-treated mice (data not shown).

### Inhibition of pAKT and induction of pro-apoptotic proteins (FOXO3a and Par-4) in WA-treated tumors

**Figure 5:**
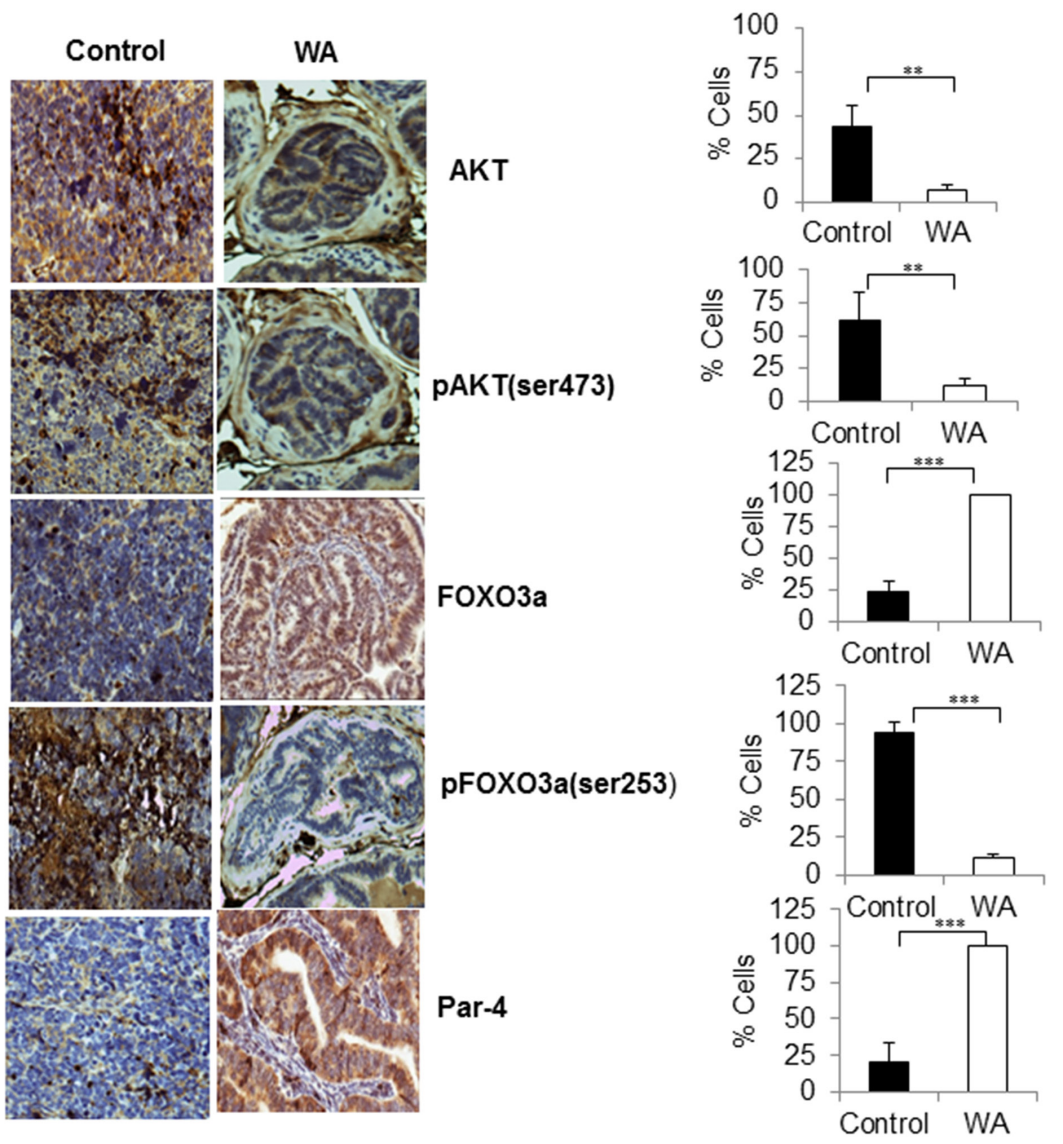
Immunohistochemical staining pro-survival, and pro-apoptotic makers in control and WA-treated prostate tumor tissues Immunohistochemical staining for AKT, pAKT, FOXO3a, pFOXO3a, and Par-4 was performed on tumor sections from control and WA-treated mice. Positive stained cells were counted and are represented as a percent.

Immunohistochemical analysis (IHC) of tumor sections showed high expressions AKT, pAKT, and pFOXO3a in control tumors, and low levels of total FOXO3a and Par-4 were seen in sections from control tumors, suggesting activation of AKT signaling in TRAMP models (Figure 5). On the other hand, induction of nuclear localization of FOXO3a and Par-4 and decreased expressions of AKT, pAKT and pFOXO3a were observed in WA-treated TRAMP tumors, confirming once again that WA inhibits AKT signaling in prostate cancer (Figure 5).

**Figure 6:**
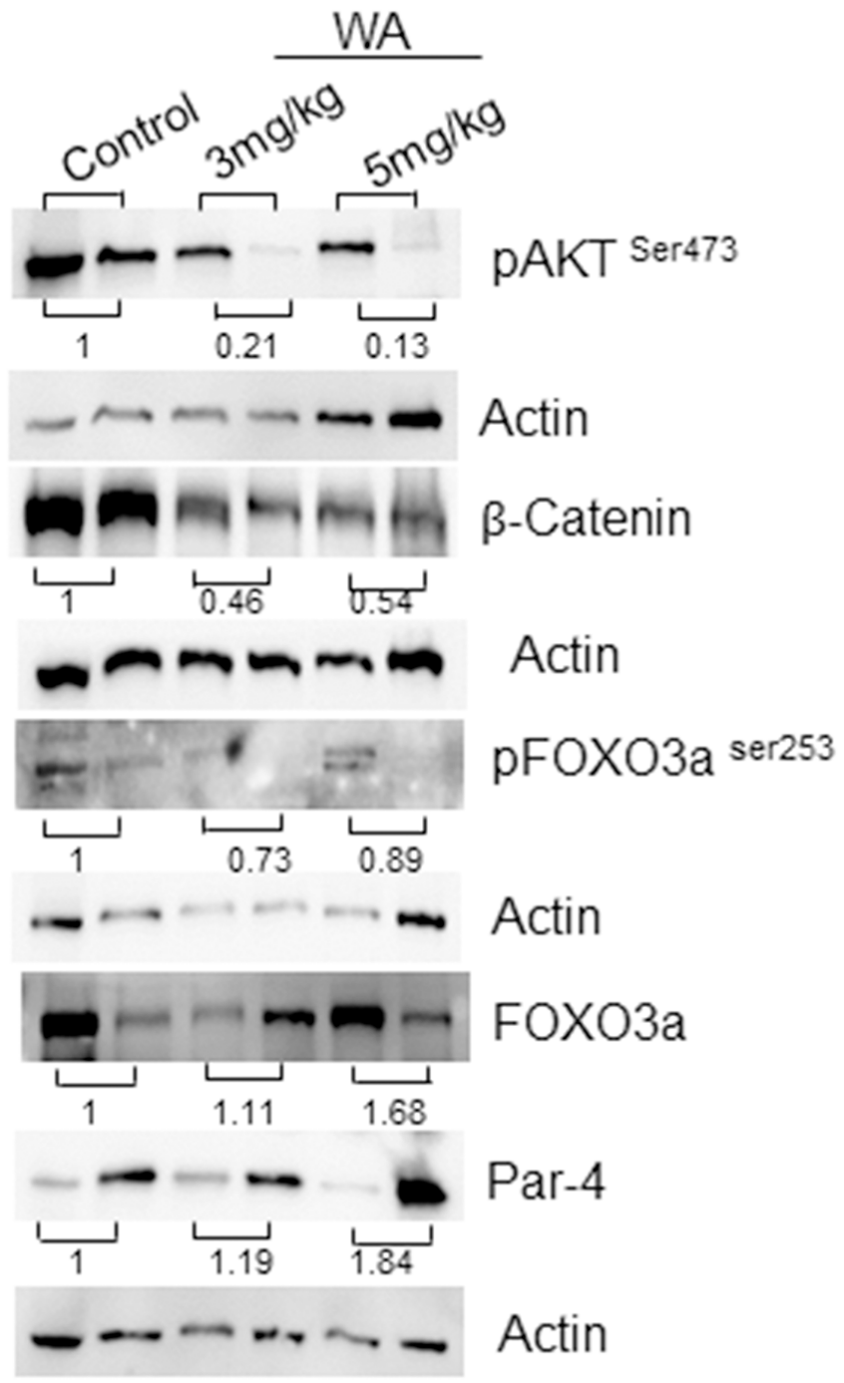
Western blot analysis in control and WA-treated prostate tumor tissues Western blot analysis was performed for AKT, pAKT, FOXO3a, pFOXO3a Par-4 , β-catenin and Actin expression in tumor tissues from control and WA-treated mice.

Next, to confirm our IHC findings, we performed western blot analysis on tumors from control and WA-treated mice. As expected, WA-treated tumors showed low expressions of pAKT and pFOXO3a when compared to control tumors. Upregulation of FOXO3a and Par-4 were seen in WA-treated tumors, suggesting abolition of AKT-mediated signaling and reversion of the pro-apoptotic function of the FOXO3a and Par-4 signaling axis in prostate cancer (Figure 6).

**Figure 7:**
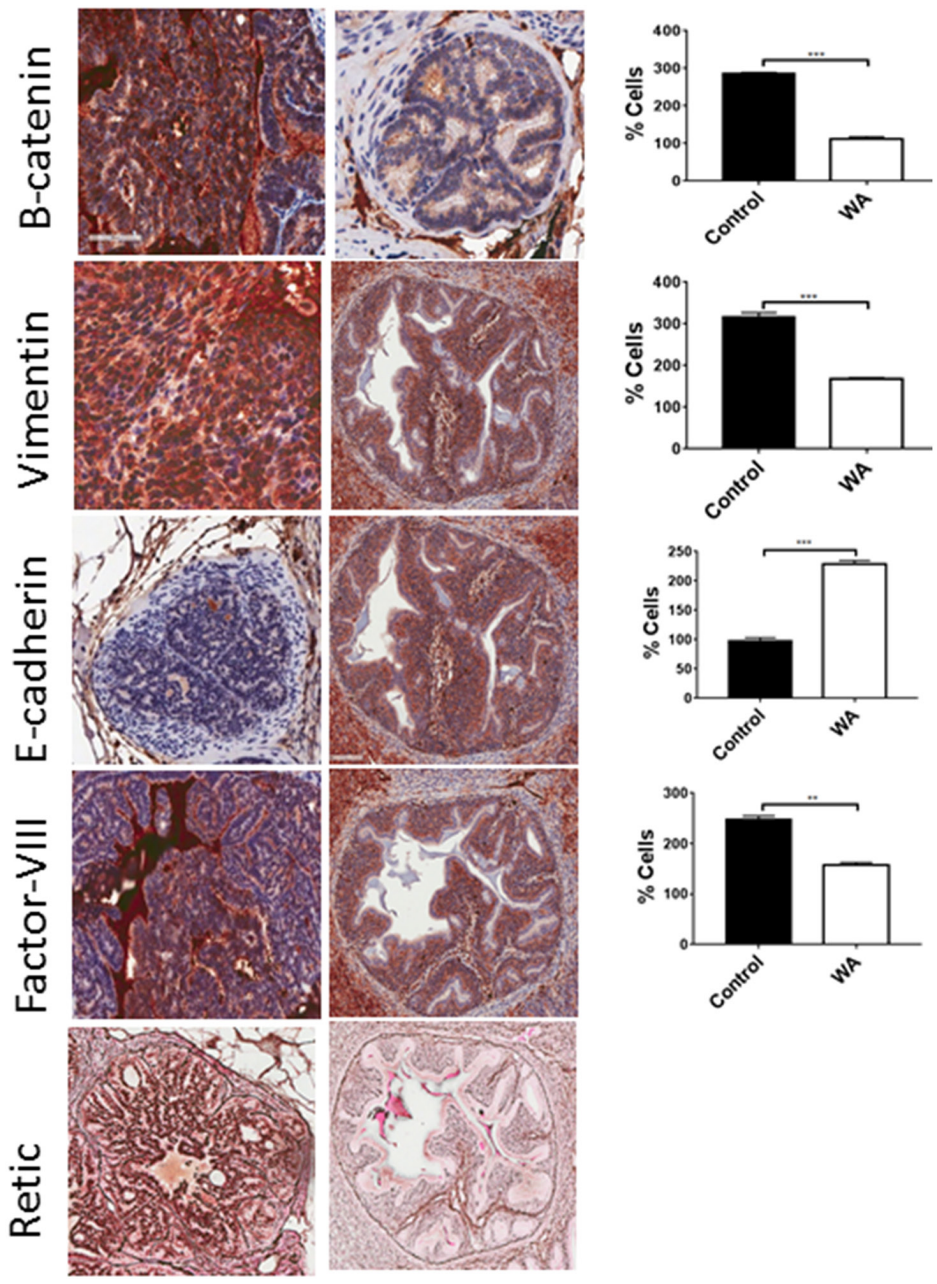
Immunohistochemical staining for EMT markers Epithelial (E-cadherin), mesenchymal (β-catenin and vimentin), and angiogenic markers (retic and factor VIII) were performed on control and WA-treated prostate tumor sections.

We found a strongly positive retic network, suggesting microvessel formation in control tumors; however, absent or lower expression in WA-treated tumor sections suggests that WA could be a potent angiogenic inhibitor. To further confirm this, we also stained with factor VIII, an angiogenic marker, and complete inhibition was found in WA-treated tumor sections, suggesting inhibition of angiogenesis (Figure 7).

## DISCUSSION

Prostate cancer (CaP) is the most commonly diagnosed cancer among men in the US, with an estimated 220,800 new cases and 27,540 deaths in 2015 alone [35]. While many treatments options are available during the initial stages of the disease, once the disease progresses to CRPC, no effective treatments are available. At this stage, clinicians resort to palliative care and extension of life. Hence, there is an immediate need for effective treatments or preventive measures to curtail prostate cancer. In this study we showed that oral administration of WA effectively suppresses the tumor burden in both primary prevention (beginning at week 6) and intervention at high-grade PIN (beginning at week 12). Hence WA, appears to be a potent compound for both chemopreventive and therapeutic effects against carcinogenesis of prostate. Additionally, WA exhibits a favorable toxicity profile as no detectable levels of toxicity were observed at the administrated doses as reflected by the lack of abnormalities in the H&E of vital organs.

Because prostate cancer is considered to be a “disease of older adults,” occurring after the age of 50 years, a chemopreventive strategy is ideal. While prostate serum antigen (PSA) screening may not be as sensitive or accurate as desired, it has routinely been used as an early detector for prostate cancer [36]. Hence, consuming dietary supplements such as WA may prevent disease progression, and it may also curtail unnecessary chemo and radiation therapies to inhibit disease progression [37]. Our results showed that oral administration of WA from weeks 6 through 34 in the TRAMP model significantly reduced tumor burden, when compared to controls. In controls, mice developed adenocarcinoma and neuroendocrine tumors beginning in week 20 and infiltrating tumors at week 29, but WA-treated tumors showed hyperdysplasia.

Several chemopreventive agents have been tested in the TRAMP model, including natural compounds such as green tea polyphenols [38, 39], genistein [40, 41], plumbagin [42], and pomegranate juice [43]. These compounds effectively arrest prostate cancer progression, and WA could be one such compound that inhibits progression either before the formation of PIN or once disease has progressed to adenocarcinoma. In our results, activation of AKT was evident in control tumors, validating previous findings [28, 44]. Ciarlo et al [45] reported that AKT activation may play an important role in the promotion of neuroendocrine tumors, a signature of the aggressive phenotype [46]. Recently, we reported that activation of AKT could be the driving force for metastasis as well as a causative factor for CRPC, because it increases pAKT expression in a stage-specific manner in human prostate cancer [10]. Hence, targeting AKT could be another option for prostate cancer treatment. While AKT activation correlates with disease progression, it is also a well-established causative factor for drug resistance in many cancer types [47–49]. As previously established, inhibition of AKT activation is the mechanism by which WA exerts an anticancer effect in prostate cancer. Using both IHC and western blot analysis in control and WA-treated tumors, this was confirmed. As expected, downregulation of pAKT expression in WA-treated tumors was seen in both sets of experiments, suggesting that AKT could be a target of WA. Inhibition of AKT by WA facilitates induction of the pro-apoptotic functions of FOXO3a and Par-4 in WA-treated tumors. Recently, Li et al [50]., demonstrated that induction of reactive oxygen species (ROS) generation, concomitant down regulation of AKT signaling and upregulation pro-apoptotic signaling resulted in combination with oxaliplatin in pancreatic cancer cells.

During the process of tumor metastasis, a cellular event termed epithelial to mesenchymal transition (EMT), is initiated and is believed to be prerequisite for tumor dissemination. We also observed inhibition of EMT markers in WA-treated tumors. Specifically, the ratio of epithelial markers (e.g., E-cadherin) and mesenchymal markers (e.g., β-catenin, snail, slug, and vimentin) may determine adhesion, polarity, and invasive characteristics of cancer cells, dictating tumor metastasis [51, 52]. We and others have reported the role of EMT in prostate cancer metastasis [53–55]. WA has been shown to downregulate EMT markers such as Vimentin and β-catenin in breast cancer stem cells [56, 57]. Oral administration of WA inhibited the expression of β-catenin, vimentin, and snail/slug (data not shown) and induced E-cadherin in prostate tumor samples, suggesting prevention of metastasis. Inhibition of lymph node metastasis was also seen in WA-treated animals; however, we could not see significant differences between control and WA-treated animals in the metastatic incidence in organs such as the liver, lung, and kidney. While the metastatic size was smaller in WA-treated animals, it is unclear why the difference in metastatic incidences between controls and WA-treated animals was not significant. Although, we found tumor inhibition at the primary site (prostate) and molecular downregulation of mesenchymal markers, yet the metastatic incidence was similar. This could be due to possibility of involvement of cancer stem cells (CSCs) that are responsible for drug resistance and metatstasis [58]. These CSCs are capable of self-renewal and can reinitiate a tumor for several generations [59]. Recently CSCs have been identified in CaP. It could be that the WA dose may not have been sufficient, or that once disease progressed (starting at week 20), the dosage should have been increased in concentration or frequency. It is worth mentioning that no metastasis was seen in up to 29 weeks with WA treatment, and most metastases were seen after week 31 (termination was at week 34 or 45). We believe that increasing WA concentration or increasing dosing frequency after 25 weeks may effectively prevent metastasis.

In conclusion, our results suggest that WA inhibits AKT activation and reverts FOXO3a-mediated Par-4 function, yielding an inhibition of prostate cancer growth in the TRAMP model. Increasing WA concentration after week 20 may inhibit micro metastasis in other organs. Finally, after nearly 39 weeks of WA treatment, no significant toxicity was found in any animals; therefore, it appears to be safe compound, and more studies may translate these findings to clinical settings.

## MATERIALS AND METHODS

Mice (C57BL/6-Tg [TRAMP] 8247Ng/J [#003135]/C56BL/6J [#000664]) were purchased from Jackson Laboratories (Bar Harbor, ME). All animals were housed under pathogen-free conditions, and the experiments were performed in accordance with the approval of the institutional Animal Care and Use Committee. Two sets of experiments were performed. In the first set, TRAMP mice received oral administration of WA [3 mg/kg, (n=8) or 5 mg/kg, (n= 13)] or vehicle (n=10) from week 6 to week 45 (39 weeks), and similarly in second set, WA 3 mg/kg body weight group consists of n=11 and vehicle group n=12 for 24 weeks. Sesame oil (vehicle) was used for the control group, and all groups received a normal diet of AIN-93M rodent food.

Mice were weighed weekly and at end of treatment, and all mice were euthanized by CO_2_ asphyxiation. Serum samples were obtained by cardiac puncture. All major organs (prostate, liver, lungs, kidney, bladder, and seminal vehicles) were dissected, and gross pathological examination was conducted for viable abnormalities. Genitourinary organs were weighed. The organs were fixed with 10% buffered formalin and subjected to H&E staining for pathological examination.

### Hematoxylin and Eosin (H&E) staining

For pathological examination of prostate tissues, H&E staining was performed according to standard laboratory protocol. For pathologic examination, H&E-stained slides were analyzed by the pathologist using light microscopy.

### Immunohistochemistry

Tumor tissue slides were dewaxed and rehydrated before antigen retrieval. They were then incubated with primary antibodies against AKT, phosphorylated AKT, FOXO3a, phosphorylated FOXO3a, β-catenin, E-cadherin, vimentin, retic and factor VIII followed by incubation with respective HRP-conjugated secondary antibodies at room temperature for 1 h. Diaminobenzidine (DAB) was used for coloration, and a dark brown color was considered to be positive staining. Positivity for staining was quantified by considering the percentage of the frozen prostate tumor tissues that were prepared using tissue extraction reagent (Life Technologies). Western blotting was performed for pAKT (ser 273), AKT, pFOX03a (ser 253), FOXO3a, and Par-4 expression in both control and WA-treated tumors. Positive bands were detected using the enhanced chemiluminescence method. Actin was used as a loading control.

### Statistical analysis

All statistics are expressed as mean ± standard deviation (SD). To calculate statistical significance, unpaired Student *t* test was used. All statistical calculations were performed using Graph Pad prism Software, (La Jolla, CA). All differences denoted by asterisks were statistically significant and were encoded in the figures (#: not significant,*P < 0.05, **P < 0.01 and ***P < 0.001).
